# P-2350. Respiratory Virus Detection among Children, Teachers, and Touch Surfaces in Childcare Centres: Preliminary Results from the DISeases TrANsmission in ChildcarE (DISTANCE) Cohort Study (2022–2024)

**DOI:** 10.1093/ofid/ofae631.2502

**Published:** 2025-01-29

**Authors:** Sheng Ye, Shuyu Deng, Bingbing Cong, Ling Guo, Tiantian Zhang, Xiaoyu Xu, Chao Shi, Xin Wang, You Li

**Affiliations:** Nanjing Medical University, NANJING, Jiangsu, China (People's Republic); Nanjing Medical University, NANJING, Jiangsu, China (People's Republic); Nanjing Medical University, NANJING, Jiangsu, China (People's Republic); Nanjing Medical University, NANJING, Jiangsu, China (People's Republic); Nanjing Medical University, NANJING, Jiangsu, China (People's Republic); Nanjing Medical University, NANJING, Jiangsu, China (People's Republic); Wuxi Centre for Disease Control and Prevention, Wuxi, Jiangsu, China; Nanjing Medical University, NANJING, Jiangsu, China (People's Republic); Nanjing Medical University, NANJING, Jiangsu, China (People's Republic)

## Abstract

**Background:**

Childcare centres are commonly regarded as high-risk settings for respiratory virus transmission. To understand transmission risks within childcare centres, we conducted a prospective cohort study that collected data on respiratory infections in children and teaching staff, contact behaviours of children, and detection of respiratory viruses on touch surfaces (Figure 1).

**Figure 1.**
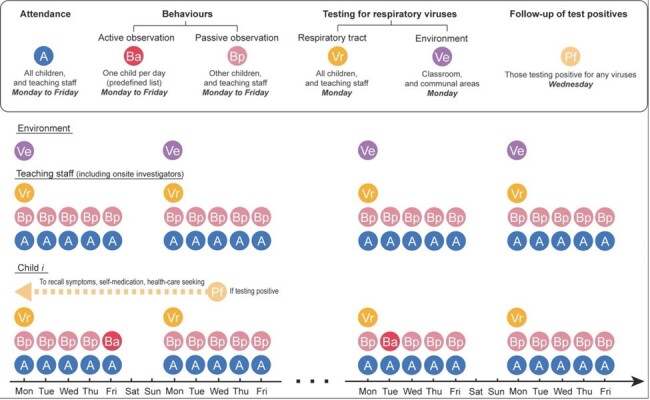
Schematic figure showing data collection and follow-up of the study.

**Methods:**

We reported updated preliminary findings based on data collected from two childcare centres in Wuxi and Nanjing, China between September 26^th^, 2022, and January 22^nd^, 2024. Weekly throat swabs were collected from 211 children and 21 teachers. 56 touch surfaces, such as desks, door knobs, and faucets, were sampled on the same day from childcare centres (Table 1). Both respiratory and surface swab samples were tested by multiplex PCR for eight respiratory viruses.

**Table 1.**
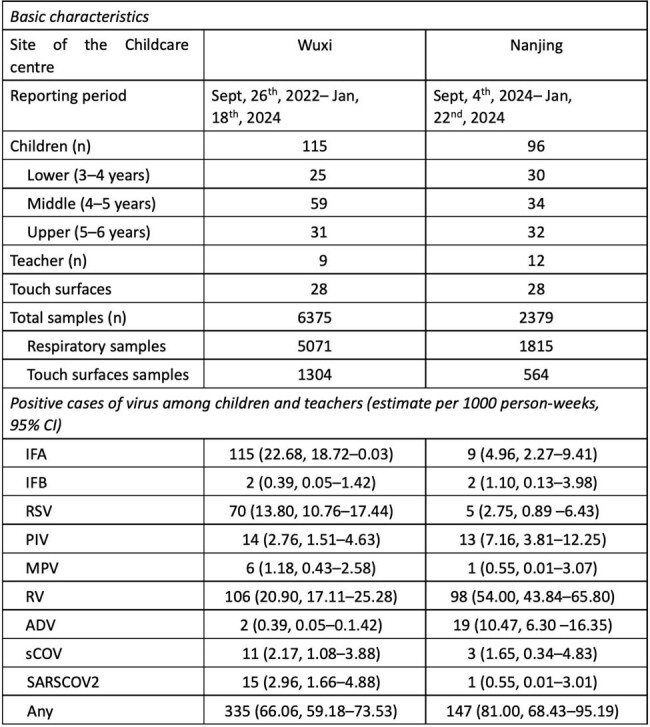
Basic characteristics of the study and Positive cases of virus among children and teachers (estimate per 1000 person-weeks, 95% CI)

**Results:**

6886 respiratory specimens and 1868 touch surface samples were collected and tested over the reporting period. Respiratory viruses were most detected among children (7.41%, 447/6032), followed by teachers (4.10%, 35/854) and touch surfaces (2.09%, 39/1868). The top four viruses detected among all children were rhinovirus, influenza A virus, RSV, and PIV (Figure 2). Specifically, rhinovirus prevailed mostly in the Nanjing site (60.11 per 1000 person-weeks, 95% CI: 48.69–73.41), while influenza A dominated in the Wuxi site (23.00 per 1000 person-weeks, 18.75–27.92). Teachers had lower viral infections than children except for influenza A, where rates were comparable: teachers (16.39 per 1000 person-weeks, 8.96–27.51) and children (18.24 per 1000 person-weeks, 14.99–21.98). For a given week, influenza A detection was correlated between students and teachers (Spearman correlation coefficient r =0.55) at the Wuxi and Nanjing sites (Table 2). However, for SARS-COV-2, there was a higher correlation between touch surfaces and individuals (children: r = 0.67; teacher: r = 0.82), than between children and teachers in Wuxi.

**Figure 2.**
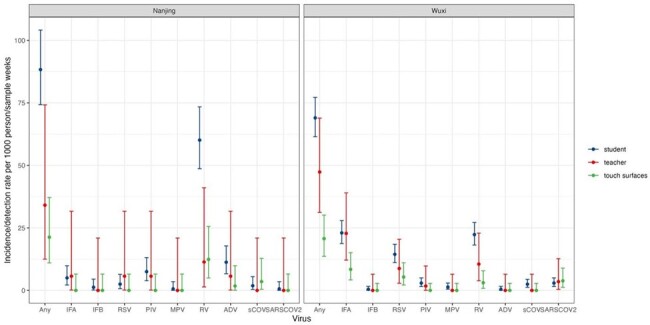
Comparison of incidence/detection rate among children, teachers and touch surfaces in two childcare centres.

**Conclusion:**

Children in childcare centres face higher risks of respiratory viral infections than teachers. There is synchrony in detecting respiratory viruses among teachers, students and touch surfaces, indicating the varied contribution of each component to respiratory virus transmission in childcare centres.

**Table 2.**
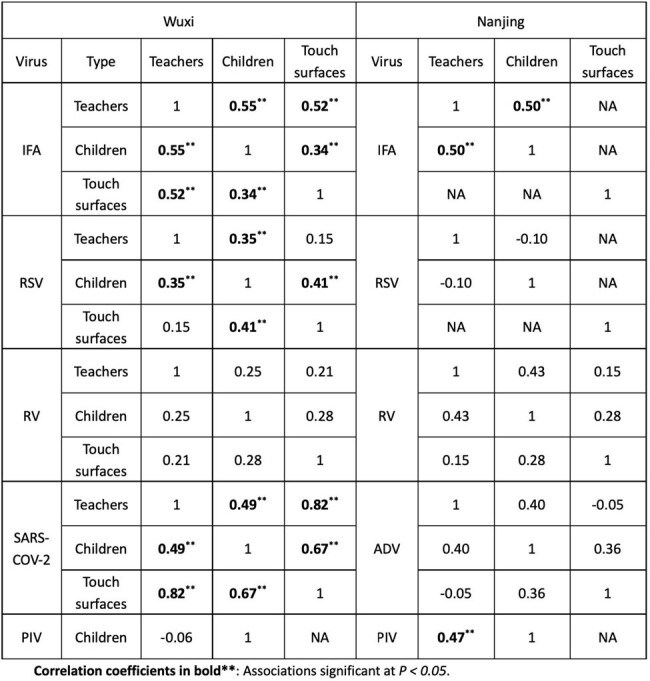
Correlation coefficients of respiratory virus detection in Nanjing

**Disclosures:**

You Li, Pfizer: Advisor/Consultant

